# Reply to Patella et al. and Lindestam Arlehamn et al.: Complex pandemic dynamics and effect of bacillus Calmette–Guérin (BCG) vaccination on COVID-19 prevalence and mortality

**DOI:** 10.1073/pnas.2017197117

**Published:** 2020-09-29

**Authors:** Carolina Barillas-Mury, Luis E. Escobar, Alvaro Molina-Cruz

**Affiliations:** ^a^Laboratory of Malaria and Vector Research, National Institute of Allergy and Infectious Diseases, National Institutes of Health, Rockville, MD 20852;; ^b^Department of Fish and Wildlife Conservation, Virginia Polytechnic Institute and State University, Blacksburg, VA 24601

In agreement with our published results (1), the epidemiological analysis by Lindestam Arlehamn et al. (2), with COVID-19 data from April 22, 2020, found a significant negative correlation (*P* = 0.0006) between mean bacillus Calmette–Guérin vaccination coverage and deaths from COVID-19. However, this correlation did not hold when the authors used updated mortality data from August 1, 2020 (2). Two major factors determine the probability that a person will die from COVID-19: first, whether the person becomes infected; second, for those who become infected, how well they respond to the infection, and this is determined by biological and social variables such as age, gender, preexisting conditions, immunological history (such as bacillus Calmette–Guérin vaccination), and the access and efficacy of medical interventions.

The dynamics of the COVID-19 pandemic are complex, and as time goes by, the degree of success of social distancing strategies predominate as the major determinant of the number of people that become infected and those that ultimately die from the infection. For example, COVID-19 mortality in the United States decreased from 6.1% on May 12 to 3.2% on August 12, 2020 (3), due, in part, to the large number of young people infected between June and August (4). We compared the mortality rates in different countries during the first 25 to 30 d of the pandemic, because the infections leading to those early deaths took place before social distancing strategies had a major impact (2). A recent independent epidemiological study also found a strong negative correlation between the years of bacillus Calmette–Guérin administration and a lower mortality over time (5).

**Fig. 1.**
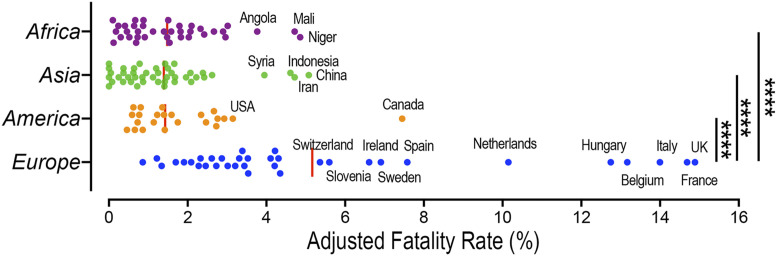
Adjusted case fatality rate from COVID-19. Countries with 1 million (M) or more citizens were included in the analysis. The percentage of positive tests was used as a proportional correction factor for the infection prevalence in those countries in which 10% or more of the tests were positive (e.g., a country in which 25% of tests were positive was considered to be underestimating the infection prevalence by 2.5-fold). The red lines indicate the mean percent adjusted case fatality; ****P < 0.0001, ANOVA. Data are from August 12, 2020 (3).

At a later stage of the pandemic, beyond the first 25 to 30 d, the case fatality rate (number of deaths/number of cases) is probably a better parameter to analyze the effect of bacillus Calmette–Guérin vaccination. However, the number of COVID-19 cases reported depends on the number of tests done and needs to be adjusted. We used the COVID-19 data from August 12, 2020 (3), to calculate the case fatality rate and the percentage of positive tests (cases detected/tests done). We find that the overall adjusted fatality rate in Europe is significantly higher than in Asia, Africa, or the Americas, with Western European countries having the highest case fatality rates (Fig. 1) (ANOVA, *P* < 0.0001).

We agree with the authors that some variables may correlate indirectly with the observed differences in COVID-19 mortality in Germany. For example, because alpine skiing is an expensive sport, it may be associated with regions with higher Human Development Index (HDI), leading to less natural exposure to tuberculosis and earlier interruption of bacillus Calmette–Guérin vaccination. Alone, the observed differences between East and West Germany would not be sufficient to draw our conclusions. However, we analyzed several different scenarios, including a coarse analysis with all countries, analysis of socially similar countries, the arrival of the pandemic to the Americans, East vs. West Germany, Eastern vs. Western Europe, and a quantitative comparison using the bacillus Calmette–Guérin index between socially similar European countries. Taken together, these multiple comparisons support the hypothesis of reduced mortality in countries with strong bacillus Calmette–Guérin vaccination programs.

The study by Patella et al. (6) and ours (1) tested different hypotheses, and also differ in the assumptions, spatial scale, and data used. Patella et al. explored whether bacillus Calmette–Guérin vaccination reduced the prevalence of COVID-19 infection reported by medical personnel in Italy using fine-scale morbidity data, while we tested the hypothesis that bacillus Calmette–Guérin vaccination reduced mortality from COVID-19, using coarse-scale mortality data. Their findings are similar to those from a previous study in Israel (7), and both studies conclude that bacillus Calmette–Guérin vaccination does not prevent COVID-19 infection. Their findings, however, do not contradict our conclusions.

We carried out a global-scale, country-level epidemiological study to explore association between bacillus Calmette–Guérin vaccination and severe COVID-19 (1). The bacillus Calmette–Guérin vaccine is thought to trigger a broad and unspecific “immune training” of the innate immune system. The innate immune system is the first line of defense for any infection, but it also identifies the type of pathogen and releases key signals to attract and direct the adaptive immune system to mount an effective and well-coordinated response. The two major components of the adaptive immune system, cellular and/or antibody-mediated immunity, are also key players to control infections, and there is no evidence that COVID-19 is an exception. One cannot assume that the training of the innate immune system in bacillus Calmette–Guérin-vaccinated individuals alone would confer complete protection from COVID-19.

We do agree with the authors in that official mortality reports may be incomplete. Nevertheless, mortality data offered clear signals of differences in COVID-19 mortality between countries. For example, a COVID-19 case fatality rate above 10%, as observed in the United Kingdom, Italy, Belgium, and the Netherlands, is unlikely to have gone undetected in any country. Analyzing COVID-19 mortality data by country by day, is more reliable than morbidity data, because case numbers are greatly influenced by the quality and quantity of diagnostic tests, criteria for testing, and the number of undetected asymptomatic cases. Strikingly, up to 45% of COVID-19 infections can be asymptomatic (8). A recent independent epidemiological study using multivariate analysis confirmed a strong negative correlation between the years of bacillus Calmette–Guérin administration and a lower mortality as the pandemic progressed over time (5).

In our study, the comparison between socially similar European countries controlled for demographic differences and for differences in HDI (1). Only countries that followed the same bacillus Calmette–Guérin vaccination scheme (universal vaccination of infants) were included (1). The duration of the protection when the vaccine is given to different age groups is not known. The authors argue that the age of vaccination does not affect the duration of the “training” of the innate immune system elicited by bacillus Calmette–Guérin, but there is no evidence to support this conclusion. We strongly agree with the authors in that if clinical trials were to demonstrate that bacillus Calmette–Guérin vaccination protects from severe COVID-19, bacillus Calmette–Guérin vaccine production should rapidly be increased to prevent the depletion of stocks currently available globally to protect children in tuberculosis-endemic areas.
